# Take the money and run? Redemption of a gift card incentive in a clinician survey

**DOI:** 10.1186/s12874-016-0126-2

**Published:** 2016-02-24

**Authors:** Jane S. Chen, Brian L. Sprague, Carrie N. Klabunde, Anna N. A. Tosteson, Asaf Bitton, Tracy Onega, Charles D. MacLean, Kimberly Harris, Marilyn M. Schapira, Jennifer S. Haas, on behalf of the PROSPR (Population-based Research Optimizing Screening through Personalized Regimens) Consortium

**Affiliations:** Division of General Medicine and Primary Care, Brigham and Women’s Hospital, 1620 Tremont Street, Boston, MA 02120 USA; Harvard Medical School, Boston, MA USA; University of Vermont, Burlington, VT USA; Geisel School of Medicine at Dartmouth and Norris Cotton Cancer Center, Lebanon, NH USA; Office of Disease Prevention, Office of the Director, National Institutes of Health, Bethesda, MD USA; Harvard T.H. Chan School of Public Health, Boston, MA USA; University of Pennsylvania and the Philadelphia VA Medical Center, Philadelphia, PA USA; Gillings School of Global Public Health, Chapel Hill, NC USA

**Keywords:** Survey methods, Provider surveys

## Abstract

**Background:**

Clinician surveys provide critical information about many facets of health care, but are often challenging to implement. Our objective was to assess use by participants and non-participants of a prepaid gift card incentive that could be later reclaimed by the researchers if unused.

**Methods:**

Clinicians were recruited to participate in a mailed or online survey as part of a study to characterize women’s primary health care provider attitudes towards breast and cervical cancer screening guidelines and practices (*n* = 177). An up-front incentive of a $50 gift card to a popular online retailer was included with the study invitation. Clinicians were informed that the gift card would expire if it went unused after 4 months. Outcome measures included use of gift cards by participants and non-participants and comparison of hypothetical costs of different incentive strategies.

**Results:**

63.5 % of clinicians who responded to the survey used the gift card, and only one provider who didn’t participate used the gift card (1.6 %). Many of those who participated did not redeem their gift cards (36.5 % of respondents). The price of the incentives actually claimed totaled $3700, which was less than half of the initial outlay. Since some of the respondents did not redeem their gift cards, the cost of incentives was less than it might have been if we had provided a conditional incentive of $50 to responders after they had completed the survey.

**Conclusions:**

Redeemable online gift card codes may provide an effective way to motivate clinicians to participate in surveys.

## Background

Clinicians provide an important and unique perspective on the health care system. Because of their integral position, clinicians perform the role of expert witnesses to contemporary medical knowledge and practice, the efficacy of health care, and the needs of the patient population. Surveys are a key way to access their ‘testimony’ and achieve better understanding of their practices and beliefs, but low response rates may limit their utility.

Previously conducted surveys have shown that medical professionals, and particularly physicians, participate in surveys at low rates that may be decreasing [1]. There are several hypothesized reasons for this, but the predominant explanation is that physicians have greater time constraints and less available time in which to take part [2]. Because clinician surveys provide such an important information source, recent studies have focused on improving participation and have identified some specific methods for doing so. Research has shown that offering financial incentives to participate up-front, as opposed to a conditional offer of an incentive after completion, increases response rate among medical professionals [3, 4]. Larger incentives yield higher response rates as well. In particular, Keating et al. found that an incentive of $50 up-front using a pre-paid check yielded a significantly larger response rate than $20 among medical professionals [5].

Providing a larger incentive before survey completion comes at a cost. With limited budgets, researchers may be hesitant to offer such incentives because this approach requires allocating money with no guarantee of a return. Particularly with the larger remunerations that have been used with doctors in the past [6, 7], dispensing money up-front can be considered a risky proposition. Pre-paid check incentives may require personal information that many providers may not want to provide, and cash or checks cannot be included with survey reminders without an increased cost. It is thus important to identify ways to administer incentives that yield a satisfactory participation rate, while simultaneously reducing the amount of potential unnecessary spending.

In this study, we examine use by participants and non-participants of a prepaid gift card incentive that could be later reclaimed by the researchers if unused and calculate the hypothetical costs of different incentive strategies. Gift cards offer several potential advantages: unlike cash or checks, they can be included with each survey reminder without an additional cost; they are logistically easier to manage at many institutions. We included a $50 gift card incentive with our electronic invitation to participate in the survey, and informed the clinicians that they had 4 months to use the code, after which the gift card codes would be canceled and the funds from any unclaimed gift cards recovered. This allowed us to potentially re-coup the money from unused gift cards. We were specifically interested in understanding whether non-respondents used the gift cards and whether there were respondents who did not redeem the cards.

## Methods

In the fall of 2014, we administered a survey to 177 clinicians, including medical doctors (MDs), doctors of osteopathy (DOs), physician assistants (PAs), and nurse practitioners (NPs) in primary care and obstetrics and gynecology (ob-gyn) at Brigham and Women’s Hospital (BWH) as part of a multi-site provider survey conducted within the Population-Based Research Optimizing Screening through Personalized Regimens (PROSPR) consortium [8, 9]. The overall aim of PROSPR is to conduct multi-site, coordinated, trans-disciplinary research to evaluate and improve cancer screening processes. The primary goal of the multi-site provider survey was to characterize women’s primary health care provider attitudes towards breast and cervical cancer screening guidelines as well as the cancer screening decision support available to the participants in their clinical practice settings [9]. Survey participants were selected if they had ordered at least 5 mammograms documented in the PROSPR database or were MDs, DOs, PAs or NPs in the primary care network at BWH. The survey was 18 printed pages long, had extensive skip logic, and required about 15 min on average to complete. Within the BWH network, we were able to obtain both emails and mailing addresses for each clinician. We thus decided to reach out with 2 email invitations for an electronic version of the survey, spaced 7 days apart. We then followed up with a mailed paper version to non-participants 7 days after the second electronic request and again 14 days later for the remaining non-participants. Our last attempt was an email invitation 14 days after the second paper survey was sent out and 42 days after the initial electronic invitation.

We purchased unique gift cards codes from an online retailer and assigned each code to a clinician. In accord with the retailer’s gift card policy in effect at the time of this study, gift card codes did not expire and were eligible for refund if not claimed. We arbitrarily chose a 4-month time period, ending on Dec 31^st^ 2014, to allow survey participants ample time to use the gift card, and when the 4 months had passed, we called the retailer and requested that the unclaimed gift card codes be cancelled and the money returned to us. If anyone used the online gift code within those 4 months, the code was considered ‘claimed’ and was no longer eligible for refund. We made up to 3 email contacts and 2 mailed contacts with each individual to request their participation in the survey and each contact included the clinician’s unique gift card code in the email invitation or paper cover letter.

We compared the rates of use of gift cards between respondents and non-respondents using chi-square tests. We also tested the association of demographic characteristics of respondents including gender, specialty, age, and survey modality with gift card redemption using a chi-square test. Finally, we estimated the potential costs of this strategy of using redeemable gift cards compared to a two hypothetical approaches: 1) Providing non-refundable gift cards or cash up-front to all 177 clinicians; and 2) providing conditional incentives only to participants who completed the survey. This study was reviewed and approved by the Institutional Review Board of Partners HealthCare.

## Results

Of the 177 recruited clinicians, 115 completed the survey for a response rate of 65.0 % (Table 1). Of the 115 respondents, 73 gift cards (63.5 %) were redeemed. Notably, only 1 person of the 62 non-respondents redeemed their gift card (1.6 %) but did not complete the survey. Women were more likely to participate than men. We found that gender, specialty, and number of contacts attempted to complete the survey were not associated with gift card code redemption among participants (Table 2). Redemption of the card was highest in providers 40 years of age or less and lowest in those over 60. We were not able to collect demographic information for those who did not participate in the survey.

**Table 1:** Use of gift cards by participation status, provider gender, and provider type (*n* = 177)

	Participated (%) *n* = 115 (65.0 %) *N* (row %)	Did Not Participate (%) *n* = 62 (35.0 %) *N* (row %)
Gift card redemption		
Redeemed	73 (98.6 %)	1 (1.4 %)
Did not redeem	42 (40.8 %)	61 (59.2 %)
Provider Type		
MDs/DOs	100 (62.9 %)	57 (37.1 %)
NPs/PAs	15 (83.3 %)	3 (16.7 %)
Provider Gender		
Male	33 (58.9 %)	23 (41.2 %)
Female	82 (68.7 %)	39 (32.2 %)

NOTE: *p*-value for the comparison of study participation by: gift card use (<,.0001), provider type (.08), and provider gender (.02)

**Table 2:** Predictors of gift card redemption among participants (*n* = 115)

Predictor	Redeemed (*n* = 73) *N* (row %)	Did not redeem (*n* = 42) *N* (row %)	Chi Square *P*-value
Provider gender			
Male	20 (60.6 %)	13 (39.4 %)	0.68
Female	53 (64.6 %)	29 (35.4 %)	
Provider age^a^			
< 40	24 (82.8 %)	5 (17.2 %)	0.02^b^
41-50	20 (57.1 %)	15 (42.9 %)	
51-60	16 (76.2 %)	5 (23.8 %)	
60+	12 (46.2 %)	14 (53.8 %)	
Specialty			
Gen. Internal Med.	2 (50.0 %)	2 (50.0 %)	0.45
Family/general practice	50 (67.6 %)	24 (32.4 %)	
Ob-Gyn	11 (50.0 %)	11 (50.0 %)	
NP/PA	10 (66.7 %)	5 (33.3 %)	
Number of contacts made^c^			
1	35 (61.4 %)	22 (38.6 %)	0.40
2	16 (76.2 %)	5 (23.8 %)	
3	6 (50.0 %)	6 (50.0 %)	
4	8 (66.7 %)	4 (33.3 %)	
5	6 (85.7 %)	1 (14.3 %)	

^a^Age was missing for 4 respondents

^b^Though there was a significant *p*-value, the data did not show any trend with regards to age

^c^There were 6 respondents that completed the survey partially and finished after a second reminder email and were thus excluded from the *p*-value calculation

We also calculated the potential costs of different incentive strategies. If we had sent out non-refundable $50 gift cards or cash up-front to all 177 clinicians, the total cost of the incentives would have equaled $8850 (Table 3). While we do not know what the response rate would have been if we had provided cash up-front instead of gift cards, the price of the 74 incentives actually claimed totaled $3700, which is less than half of the initial outlay. Furthermore, since some of the survey respondents did not redeem their gift cards, the cost of survey incentives was less than it might have been if we had provided a conditional incentive of $50 to responders after they had completed the survey. Thus, had we used a conditional incentive approach, and the response rate was the same as observed, the cost would have been $5750.

**Table 3:** Potential Cost of Different Incentive Strategies

Method:	Description	Estimate of # participants receiving an incentive payment	Total cost
(1) Up-front incentive	Gift cards sent out with study invitation without reclaiming unused funds or use of cash with study invitation	177	$8850
(2) Up-front incentive with return of un-redeemed cards	Gift cards sent out with study invitation and funds reclaimed if not redeemed within 4 months	74	$3700
(3) Conditional incentive	Gift cards sent out only after completed survey is returned by participant	115	$5750
	Potential Savings for Method 2 vs. Method 1		$5150
	Potential Savings for Method 2 vs. Method 3		$2150

## Discussion

Financial incentives are an important tool to improve response rates in surveys of health care providers [1, 3, 4]. Up-front incentives, as opposed to conditional incentives, have been shown to be an important approach [3–5], but are risky as funds are expended without the guarantee of achieving the desired response rate. We explored a way to circumvent this problem with the use of online gift card codes that could be refunded if they were not used. We examined the risk of eligible participants using the gift card codes without completing the survey, and the potential tradeoff between improved response rate and up-front remuneration. Our results suggest that the risk of this approach is very small. Of our survey population, only 0.6 % took the money without participating (1 out of 177). Seemingly, the medical professionals in this sample didn’t try to take the money and run. We also found that use of redeemable gift card codes were less expensive than it would be to provide a cash (non-redeemable) incentive.

There may be several potential explanations for our study findings. One could hypothesize that the providers who weren’t going to participate on principle or due to time constraints, didn’t bother to read the invitation letter thoroughly. In contrast to cash, where the bills fall out of the envelope, people who didn’t take the time to open the email or letter and at least skim it, would not know there was the potential for a free gift card. Additionally, the effect could also be due to the fact the survey was sent out by a colleague within their own medical system and so the participants may feel some personal accountability. Because of this, the generalizability of our findings to other settings should be confirmed.

Interestingly, a notable proportion of health care providers who participated didn’t redeem the gift card. Only 63.5 % of the survey completers cashed in their reward (73 out of 115). There are several potential reasons for this: they could have been participating for altruistic purposes and thus didn’t need to take the money, they could have skipped the invitation letter and not realized there was a gift card included, or they could have planned to use the code at a later date and simply forgotten it was there. Despite the explanation, we were able to re-gain these funds that may otherwise have gone to waste. This combination of honesty and altruism lead to reduced survey cost, while maintaining the benefits of having offered a respectable incentive up-front.

In addition to generalizability, an additional limitation is that we did not use a randomized design to test the cost effectiveness of different incentive structures. This survey was deployed primarily to maximize the response rate of the data collection rather than to compare methods. For the cost analysis, we assumed that we would have observed equal recruitment with redeemable gift cards or cash or conditional response approach; it is possible that the response rate could have differed with these scenarios.

## Conclusions

This up-front gift card code approach took advantage of the proven strategies for survey administration to increase response rate. The $50 up-front gift card incentive enabled the recruitment of those who needed an incentive to participate, but also allowed us to capitalize on those who participated for more altruistic reasons. This approach allowed us to use a higher incentive value up-front. Additionally, the use of gift card codes allowed us to include remuneration with every participant contact, which cannot be done with cash or check. We found that only one person used the gift card without participating, and that our incentive costs ended up being lower than they would have been if we had even been able to get the same response rate with conditional incentives. This methodology could be a cost-effective way for others to get higher participation rates from health care providers, a group that has been hard to recruit without the use of a financial incentive.
